# Patient Health Questionnaire-9 versus Edinburgh Postnatal Depression Scale in screening for major depressive episodes: a cross-sectional population-based study

**DOI:** 10.1186/s13104-016-2364-0

**Published:** 2017-01-21

**Authors:** Iná S. Santos, Beatriz Franck Tavares, Tiago N. Munhoz, Patricia Manzolli, Gisele Bartz de Ávila, Eduardo Jannke, Alicia Matijasevich

**Affiliations:** 1 Federal University of Pelotas, Rua Marechal Deodoro, 1160, 3o andar, Pelotas, 96020-220 Brazil; 2 Department of Mental Health, School of Medicine, Federal University of Pelotas, Pelotas, Brazil; 3 Department of Preventive Medicine, School of Medicine, University of São Paulo, São Paulo, Brazil

**Keywords:** Patient Health Questionnaire-9, Edinburgh Postnatal Depression Scale, Major depressive episode, Screening, Accuracy

## Abstract

**Background:**

Major depressive episodes (MDE) are frequent at the population level and are generally associated with severe symptoms that impair performance of activities of daily living of individuals suffering from this condition. The aim of this study was to compare the accuracy of two tests that separately showed suitable properties in screening for MDE: the Patient Health Questionnaire-9 (PHQ-9) and the Edinburgh Postnatal Depression Scale (EPDS).

**Methods:**

In a previous study, the sensitivity and specificity of the PHQ-9 and the EPDS in screening for MDE were compared with a structured diagnostic interview conducted by psychiatrics and psychologists using the Mini International Neuropsychiatric Interview as the gold standard. In a sample of adults living in the community in Pelotas, Brazil, the PHQ-9 and EPDS were applied at the same interview and the gold standard on a median of 17 days later. The interviews were carried out at the participant’s home.

**Results:**

447 Individuals (191 men and 256 women) were assessed. The PHQ-9 and the EPDS results were concordant in 87.5% of the respondents, with a moderate agreement beyond what was expected by chance alone (kappa = 0.61). The areas below the ROC curves were not statistically different (82.1% for PHQ-9 and 83.5% for EPDS) (p = 0.291), thus indicating that the two tests had similar moderate accuracy.

**Conclusions:**

PHQ-9 and EPDS may be applied with equal confidence in screening for MDE in the community.

## Background

Major depressive episodes (MDE) are frequent at the population level and are generally associated with severe symptoms that impair performance of activities of daily living of individuals suffering from this condition [1, 2]. Worldwide, population-based surveys that included more than 37,000 individuals living in ten countries on four continents have recorded lifetime prevalence of MDE ranging from 8 to 12% [3].

The age of depressive symptoms onset is in the middle 20s, with the peak risk period for onset ranging from mid-late adolescence to early 40s [1, 3]. The approximately twofold increase in risk of depression among the women in comparison to the men is consistent over cultures and most age groups [4]. Conjugal situation (more frequent among individuals who are unmarried and live without a partner) and family genetic factors (parental depression increases the risk of the offspring also developing depressive episodes) are recognized risk factors for MDE [1, 3]. Epidemiological studies using a range of different methods have documented higher overall rates of depression over time, with increasing rates in the young, associated with a shift forward to younger ages [1]. Although older individuals may be less likely to recognize depression as a mental disorder, and hence are less likely to remember depressive episodes as such, or to report these episodes in interviews on mental health [5], the evidence that rates of depression in the elderly are higher compared with those observed in past studies suggest the presence of a cohort effect.

A number of adverse consequences of major depression have been described. School failure, low probability of ever marrying or higher probability of early marital timing and divorce, teen childbearing, negative parenting behaviors, work absenteeism, lower income-earnings, comorbidity and elevated risk of early death are all associated with major depression [1].

Recognition of depression as a public health problem has led to creation of a variety of screening instruments for use in research and in primary healthcare services, with the aim of identifying individuals at risk of MDE, at an earlier stage [6]. Although two meta-analyses [7, 8] and a quasi-experimental study [9] found no evidence of effectiveness of screening at primary healthcare services, the availability of reliable and valid information is essential for estimating and monitoring depression prevalence and time trends in depression prevalence by means of epidemiological research at the population level.

Because the properties of the screening tests vary as a function of the socio-demographic and cultural characteristics of the population to which the tests are applied, it is recommended that their use should be preceded by studies that evaluate these properties within the context in which they will be used [10]. The sensitivity and specificity of two depression screening instruments, the Patient Health Questionnaire-9 (PHQ-9) [11] and the Edinburgh Postnatal Depression Scale (EPDS) [12], were evaluated on a single sample of adults living in the city of Pelotas, RS, Brazil, and the results have already been published [13, 14], The objective of the present study was to compare the accuracy of the two tests, which separately showed good sensitivity and specificity in screening for MDE among adults living in the community.

## Methods

A cross-sectional population-based study was conducted in the urban zone of the municipality of Pelotas between February and June 2012 to evaluate the health of adolescents, adults and elderly people. A sampling design of two-stage conglomerates with probability proportional to size was used. According to the 2010 Population Census there were 495 census tracts, the primary sampling units. The secondary sampling units were households. All private households with permanent resident as of December 2011 in the 130 census tracts randomly selected were listed. In each census tract drawn, around 12 households were randomly selected for the survey. All the people living in the households drawn who were 10 years of age or over were eligible. The participants were interviewed at home, by trained interviewers, through applying a structured questionnaire that included questions about their economic condition, schooling, marital status, skin color, occupation, health, and behavior. The adults (≥20 years of age) answered the PHQ-9 and EPDS questionnaires, and these were applied by general interviewers. Individuals who had cognitive or mental disabilities confirmed by the fieldwork supervisor, as well as those institutionalized (hospitals, elderly homes, among others), were excluded.

Validation studies on PHQ-9 [13] and EPDS [14] were conducted on a subsample of adults (≥20 years of age). The sampling process for the validation studies was conducted weekly, starting from the interviews that were conducted for the main study. Through simple random draws, one-third of the households included in the main study were selected for the validation studies. The person in charge of the draw was unaware of the results from the PHQ-9 or EPDS tests that were applied in the main study. In each household thus selected, all the people living there who were 20 years of age or over, independently of the PHQ-9 or EPDS scores, were invited to receive a second visit for a supplementary interview. This second interview was conducted by a mental health professional (psychiatrist, psychologist or medical resident in psychiatry), who had previously been trained to apply and interpret the gold-standard instrument and was blind to the scores achieved by the participant in the PHQ-9 and EPDS questionnaires. The participants were unaware of the professional training of these interviewers, so that this would not influence the responses.

The PHQ-9 consists of nine questions that assess the presence of each of the symptoms of MDE, as described in the Diagnostic and Statistical Manual of Mental Disorders (DSM-IV) [15] (depressed mood; loss of interest or pleasure in doing things; problems relating to sleep, tiredness or lack of energy; changes in appetite or weight; feelings of guilt or uselessness; problems of concentration; feelings of being slow or restless; and suicidal thoughts). The frequency of each symptom over the preceding 2 weeks was evaluated on a Likert scale from 0 to 3.

The EPDS was originally constructed to identify postpartum depression, but it can be applied to screen for depression in the community, including among men [16]. The EPDS consists of a scale of 10 items, each with four possible responses from 0 to 3, which express the intensity of depressive symptoms over the 7 days preceding the interview.

The questionnaire for the first interview (main study) was set up in sections. The PHQ-9 was applied after the participants had answered the questions in the sections relating to socio-demographic factors, behavioral factors, chronic diseases and use of medications. Following the PHQ-9 application, there were questions on the subjects’ use of and access to healthcare services and their dietary habits, and then the EPDS was applied. Further details on the methodology of the validation studies for the PHQ-9 and EPDS, along with the Portuguese-language versions used, can be obtained in other published papers [13, 14].

To calculate the sample size, the following parameters were used: sensitivity and specificity of 80%, acceptable error of 10% points upwards or downwards and significance level of 95%. Thus it was necessary to include around 200 subjects with MDE and 200 without MDE. Given that the point-prevalence of depressive symptoms among the adult population of Pelotas had been found to be around 30% [17], it was estimated that with a sample of around 600 individuals, it would be possible to locate around 200 with MDE.

The gold standard used was the Mini International Neuropsychiatric Interview (MINI) [18], which has been validated for use in Brazil [19]. This structured diagnostic questionnaire assesses the presence of mental disorders, in accordance with DSM-IV and ICD-10. For depressive disorders, it has sensitivity and specificity of 92% [19]. In the present study, the gold standard was used to diagnose the presence of MDE. All individuals who were considered to be positive for MDE gave responses to an additional group of questions that investigated other possible causes for the symptoms, such as direct effects of substances, organ disorders, medical illness or presence of psychotic symptoms, or whether the symptoms would be better explained as reactions to grief, for which the diagnosis of MDE would be rejected.

The data analysis included calculation of the sensitivity and specificity for each score on a continuous scale for each of the tests. For each PHQ-9 and EPDS cutoff point, the sensitivity (proportion of individuals with MDE according to MINI criteria that were correctly identified by the test), specificity (proportion of individuals without MDE according to the gold standard correctly identified as such by the test), positive predictive value (proportion of true positives among all positives identified by the test), accuracy (proportion of true positives and true negatives identified by the test), and positive likelihood ratio (the odds that the given cutoff point would be expected in an individual with in opposed to one without MDE according to the gold standard) with 95% confidence intervals were calculated.

To compare the accuracy of the tests for identifying individuals at risk of MDE, the sensitivity and 1-specificity values of each of the cutoff points for the PHQ-9 and EPDS were plotted on a single receiver operating characteristic (ROC) curve. The cutoff point with greatest sensitivity and specificity on the ROC curve was defined as the lowest value for the equation {(1 − sensitivity)^2^ + (1 − specificity)^2^}. The accuracies of the PHQ-9 and EPDS were compared by means of the areas under the respective ROC curves. The concordance between the two tests, i.e. beyond what would be expected by chance, was calculated by means of the kappa statistic.

The main study and the validation studies were approved by the Research Ethics Committee of the School of Medicine, Federal University of Pelotas, in accordance with protocols 77/2011 and 14/2012, respectively. A free and informed consent statement was signed by each participant in the main study before information was gathered. The individuals who were diagnosed as positive through the gold-standard assessment were attended at home and/or were referred to the healthcare services.

## Results

A total of 533 individuals were identified as candidates for the gold-standard interview. Of these, 447 (84%) were assessed: there were 29 refusals; 51 were deemed to be losses after three attempts to find them; and six were found to have moved away from this municipality. The interviews with mental health professionals (gold standard) were held on average 24 days after application of the two tests (median of 17 days).

The individuals evaluated comprised 191 men and 256 women. The majority (83.2%) were under the age of 60 years and 76.5% self-declared as having white skin color. With regard to socioeconomic variables, more than one-third (39.7%) were living in families with mean monthly incomes ≤3 minimum wages and 15.3% had only attended school until the fourth year of elementary school. More than half of the participants were doing paid work at the time when the PHQ-9 and EPDS were applied (58.8%) and were living with partners (65.5%).

The individuals who were lost from the gold-standard interview were similar to those who were interviewed by the general interviewers, with regard to all the characteristics investigated, except in relation to paid work. The frequency of unemployment among individuals evaluated by means of the gold standard was greater than when the PHQ-9 and EPDS were applied (respectively, 60.4 and 41.2%).

**Fig. 1:**
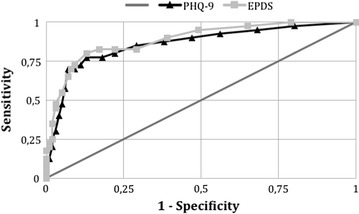
Receiver operating characteristic (ROC) curves of the Patient Health Questionnaire (PHQ-9) and Edinburgh Postnatal Depression Scale (EPDS) for screening for major depressive episodes among adults living in the community. Areas under the ROC curve: PHQ-9 = 0.821; EPDS = 0.835

The gold-standard interview identified 40 individuals (32 women and eight men) presenting MDE (8.9%; 6.3–11.6%). For the PHQ-9, values ≥9 were more accurate for identifying individuals at greater risk of presenting MDE (Fig. 1). At this point, the sensitivity was 77.5% (61.5–89.2%), specificity 86.7% (83.0–89.9%), positive predictive value 36.5% (26.3–47.6%), and positive likelihood ratio 5.8 (4.3–7.9) (Table 1). A total of 85 individuals (19.0%) scored ≥9. The area under the ROC curve indicated a general test accuracy of 82.1%.

**Table 1:** Properties and 95% confidence intervals of the Patient Health Questionnaire-9 (PHQ-9) and Edinburgh Postnatal Depression Scale (EPDS), at the cutoff points of maximum sensitivity and specificity for screening for MDE among adults in the community

Instrument	Sensitivity	Specificity	PPV	PLR
PHQ-9 ≥ 9	77.5% (61.5–89.2)	86.7% (83.0–89.9)	36.5% (26.3–47.6)	5.8 (4.3–7.9)
EPDS ≥ 8	80.0% (64.4–90.9)	87.0% (83.3–90.1)	37.6% (27.4–48.8)	6.1 (4.6–8.3)

*MDE* major depressive episode, *PPV* positive predictive value, *PLR* positive likelihood ratio

For the EPDS, the best cutoff score was ≥8 (Fig. 1), and this value was reached by 85 individuals (19.0%). The crude concordance between the two tests was 87.5%, with a moderate kappa value (0.61). The sensitivity of EPDS ≥8 was 80.0% (64.4–90.9%), specificity 87.0% (83.3–90.1%), positive predictive value 37.6% (27.4–48.8%), and positive likelihood ratio 6.1 (4.6–8.3) (Table 1). The area under the ROC curve showed a general accuracy of 83.5%, i.e. similar to that of the PHQ-9 (p = 0.291).

## Discussion

The areas under the curves, and also their formats, indicated that the PHQ-9 and EPDS presented similar and moderate accuracy with regard to identifying adults living in the community who were at greater risk of presenting MDE. Such a finding is in line with the results of a systematic review planned to examine the accuracy of depression screening instruments (including PHQ-9 and EPDS), which concluded that no single instrument was superior to another [20].

In the current study, the two tests were concordant in 391 (87.5%) of the 447 respondents. Good concordance between the tests was seen even with symptom recall times for the two scales differing by 1 week. Similar level of agreement (83%) was reported by Yawn et al. [21] in a study specifically planned to compare the PHQ-9 and EPDS as screening tools for postpartum depression. The natural history of the depression may have contributed towards the comparability of the two tests, given that once manifested, the depressive symptoms tend to persist for weeks (with a median duration of 3 months) and, in 20% of the cases, they remain chronic for 2 years or more [22].

Out of the 85 individuals who were screened positive through the PHQ-9, 57 (67.1%) were also positive according to the EPDS. Among the 56 individuals for whom the two tests had discordant results, the gold standard indicated that in half of the cases, the PHQ-9 result was correct and in the other half, the EPDS result. Further work is required to identify reasons for disagreement.

The positive likelihood ratios, both for the PHQ-9 and for the EPDS, were around six, thus indicating that results from these tests that are ≥9 and ≥8, respectively, are six times more likely to occur among individuals with MDE, in consultations with mental health professionals, than among individuals without MDE. The positive predictive values for the two tests were also very similar (36.5%; 26.3–47.6% for PHQ-9 at the cutoff ≥9; and 37.6%; 27.4–48.8% for EPDS ≥8). Thus, if these two tests are applied for population screening, they will be equally efficient: two in every five individuals with positive screening through either of the tests will present MDE.

Among the limitations of this study, 16% of individuals could not undergo the gold standard interview and, though, were not included in the validation sample. They were similar to those included in the sample regarding all socioeconomic, demographic and behavioural characteristics investigated, except to be employed. The prevalence of PHQ-9 ≥9 and EPDS ≥8 among people that failed to be included in the validation sample was similar to those included in the sample (22.1 vs 19.0%, p = 0.510 for PHQ-9; and 24.4 vs 19.0%, p = 0.250, for EPDS, respectively). It looks like the loss of these individuals may not have impaired the sensitivity estimation in the present study. Additionally, in regard to ethical aspects, neither the lost individuals nor those that refused to attend the gold standard interview had replied positively to the questions on risk of suicide of both the PHQ-9 and EPDS questionnaires.

Both the PHQ-9 and the EPDS were applied in the middle of the interview. There were questions on the subjects’ use of and access to healthcare services, as well as on their dietary habits between the PHQ-9 and the EPDS application. However, the fact that both tests were applied to the same sample, in the same interview and in sequence may have jogged the interviewees’ memories with regard to answering the questions of the EPDS, in comparison with the PHQ-9, given that the PHQ-9 was applied first. It is possible that this may have introduced some information bias.

Another limitation was the gap of about 17 days (median) between the PHQ-9 and EPDS application and the gold standard administration. Because the validation studies were nested within a large epidemiological study with a complex logistics, a delay on the execution of some of the implementation steps was difficult to prevent. It is possible that depressive symptoms may have changed over this period due to two main reasons. First, the PHQ-9 and EPDS were designed to enquire about feelings over the last fifteen and the last 7 days, respectively. Second, the duration of the MDE may vary with age and with the natural history of the disorder [4]. Recurrent depression typically has shorter episode duration. The young have more frequent episodes of shorter duration whereas the elderly has long episodes and chronic depression. However, the reported median duration of MDE in the community is 3 months [23], what may have minimized at least in part the negative effect of the time lag over the observed sensitivity of the PHQ-9 and EPDS.

Despite the standardization procedures undertaken before the study initiation, another flaw is the lack of assessment of the inter-rater reliability of the gold-standard evaluators.

Finally, there are concerns that EPDS is not suitable for men because it detects distress but not necessarily depression [24], and that EPDS has a different factor structure in men [25–28]. The EPDS, as much of the mental screening instruments assess for common mental disorders including anxiety, depression and psychological distress [20]. In the current study the gold standard interview was planned to identify true cases of MDE so that all cases that scored positive at the screening due to anxiety or psychological distress were classified as false positive results. According to the gold-standard, only eight of the 191 men included in the study presented a MDE. Such a small prevalence prevented us from conducting separate analyses according to the sex of the participants.

## Conclusion

In localities with socioeconomic, demographic and morbidity profiles similar to those of the city of Pelotas, both the PHQ-9 and the EPDS can be used confidently for screening for MDE in the community. Both of these tests have the advantage of containing few questions (nine and ten, respectively) and only taking around 5 min for application among adults [13, 14].

## Abbreviations

MDE: major depressive episode
PHQ-9: Patient Health Questionnaire
EPDS: Edinburgh Postnatal Depression Scale
PPV: positive predictive value
PLR: positive likelihood ratio
ROC: receiver operating characteristic
